# Thick calcification from a GIST of the stomach penetrating into pericolic soft tissue - report of a case

**DOI:** 10.1186/1477-7819-9-45

**Published:** 2011-04-29

**Authors:** Cheng-Chan Yu, Cheng-Chung Wu, Jen-I Hwang, John Wang, Chi-Sen Chang

**Affiliations:** 1 Department of Surgery, Taichung Veterans General Hospital, No. 160, Sec. 3, Taichung-Kang Rd., Taichung, 40705, Taiwan; 2 Department of Radiology, Taichung Veterans General Hospital, No. 160, Sec. 3, Taichung-Kang Rd., Taichung, 40705, Taiwan; 3 Department of Pathology, Taichung Veterans General Hospital, No. 160, Sec. 3, Taichung-Kang Rd., Taichung, 40705, Taiwan; 4 Department of Medicine, Taichung Veterans General Hospital, No. 160, Sec. 3, Taichung-Kang Rd., Taichung, 40705, Taiwan

## Abstract

Thick calcification is a rare presentation of gastrointestinal stromal tumor (GIST). Penetration into gastric mucosa and pericolic soft tissue has never been reported. We report a case of gastric GIST with cystic degeneration and thick calcification in an 81-year old female, who presented with hematemesis and severe abdominal pain. Thick calcification of this tumor penetrating into pericolic soft tissue was noted and successfully treated by distal gastrectomy and partial colectomy. For gastrointestinal tumors with thick calcification, even with benign behavior, surgical intervention should be considered for both oncological considerations and prevention of catastrophes like perforation or penetration into surrounding soft tissue.

## Introduction

Calcification within primary gastrointestinal stromal tumor(GIST) has been reported[1-3], but thick calcification within GIST is rare[1]. Thick calcification of a GIST penetrating into surrounding soft tissue has never been reported. Herein, we report the first case of thick calcification from a gastric GIST with cystic degeneration penetrating into pericolic soft tissue.

## Case presentation

**Figure 1:**
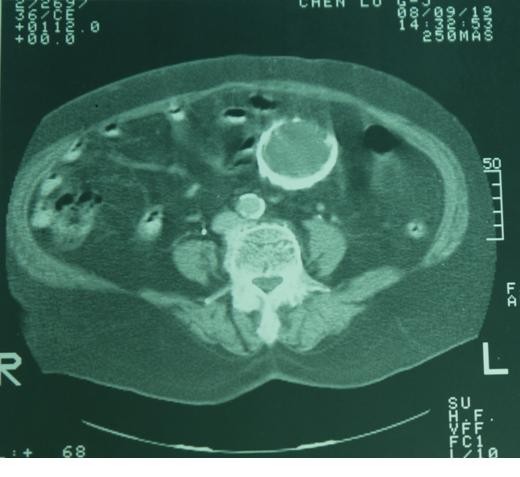
**Gastric mass with circumscribed calcification was found by the CT scan 2 years ago**. Greater thickness over the dependent part was noted.

An 81-year-old female with hypertension and gout was admitted to Taichung Veterans General Hospital due to abdominal pain and hematemesis. She began to suffer from intermittent epigastralgia more than 10 years ago, and a 4 cm gastric tumor was found. The abdominal pain got worse 2 years before admission, and she went to a local hospital where abdominal CT scan revealed a gastric tumor about 6 cm in length with well-circumscribed calcification(figure 1). Surgical intervention was suggested, but she declined. About 10 days before admission, tarry stool passage was noted, and bloody vomitus was found 1 day later. UGI scope revealed submucosal gastric tumor with central ulceration and she was then transferred to our hospital.

**Figure 2:**
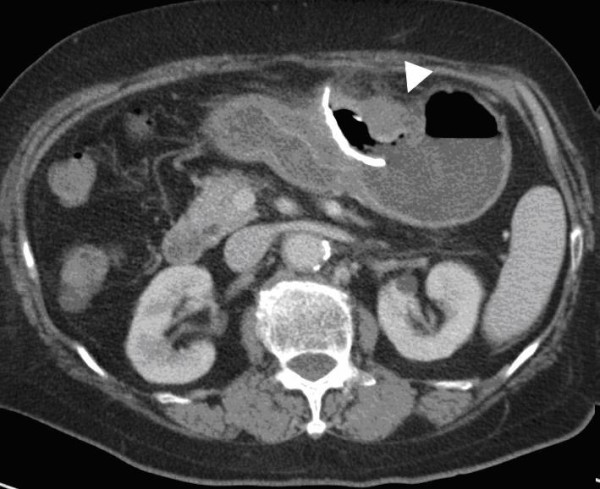
**Pre-operative CT scan showed gastric mass with curvilinear calcification penetrating into surrounding soft tissue**. Localized abscess formation was noted. A gastric tumor adjacent to the calcification was noted.

**Figure 3:**
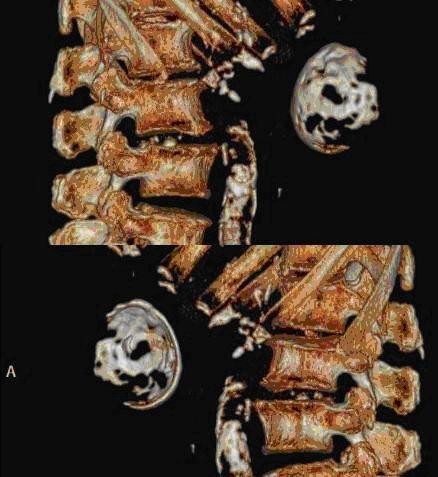
**3D CT reconstruction showed an unusual semicircular calcified plate in the upper abdomen**. (upper: Right side view, lower Left side view) Ingested foreign body was excluded by history and large size of the calcification.

**Figure 4:**
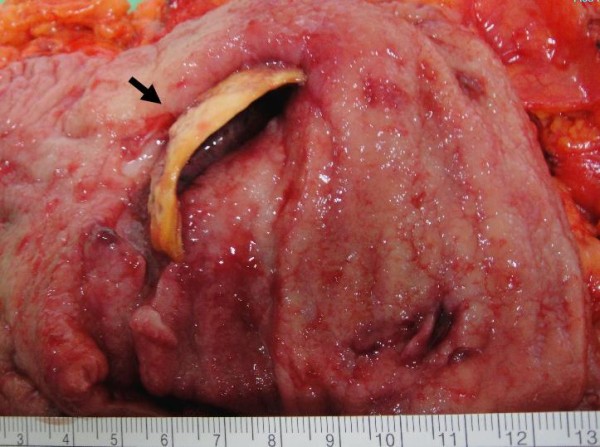
**Thick calcification with deep ulceration (black arrow) penetrating into pericolic soft tissue with abscess formation was noted**.

**Figure 5:**
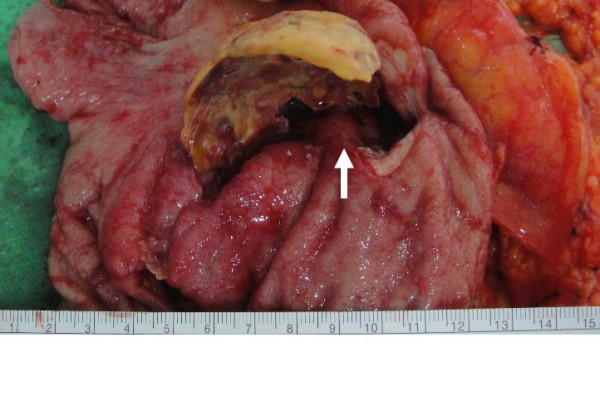
**Gastric tumor(white arrow) adherent to the calcified plate was also noted**.

Physical examination showed upper abdominal tenderness with mild muscle guarding. The plain radiography showed an irregular shape calcification over upper abdomen. UGI scope revealed deep gastric ulcer with foreign body. CT scan showed an irregularly shaped space-occupying lesion in front of the stomach with plate calcifications and localized free air (figures 2 and 3). Under the impression of perforated gastric tumor, emergent laparotomy was performed. An infiltrative mass between the stomach and transverse colon was noted during operation. A sharp, bone-like and thick calcified plate penetrating into the gastric mucosa and pericolic soft tissue was observed. A submucosal tumor about 2.3 cm in size adherent to the calcified plate was also noted (figures 4 and 5). Distal subtotal gastrectomy and partial colectomy were performed. The patient was discharged 13 days after operation uneventfully. Microscopically, spindle-shaped tumor cells with low mitotic frequency (4/50 HPF) were found. Immunohistochemical staining of the tumor demonstrated diffusely strong positive reactivity for CD 117, positive reactivity for CD34, but negative reactivity for S100 protein and desmin. The diagnosis of the tumor was established as GIST. Due to the small size and the paucity of mitotic figures of the tumor located in the stomach, it was classified as very low risk[4]. Sporadic GIST was impressed due to no family history of GIST nor other GIST presented in this patient.

## Discussion

GISTs are uncommon tumors originating from the interstitial cells of Cajal, which are pacemaker cells regulating autonomic motor activity in the gastrointestinal tract. The majority of the GISTs arise in the stomach (60-70%), followed by small intestine, colon, esophagus, omentum, and mesentery[2,3]. The tumor size, mitotic figures and the organ of origin determine the biological behavior of GIST[4]. Patients with GIST may present with abdominal pain, satiety, obstruction, or GI bleeding. However, thick calcification of a GIST penetrating into pericolic soft tissue has never been reported.

CT is considered to be the imaging modality of choice for the detection, staging, surgical planning, and follow-up of patients with GIST[5,6]. Most tumors are seen as well delineated soft tissue masses with heterogenous contrast enhancement. Necrosis, calcification, and ulceration are most commonly seen in large tumors that present a more aggressive behavior[6].

Focal calcification within GIST has been reported, ranging from 10% to 50% in reported series[6-8]. However, extensive thick calcification visible on plain radiograph is a rare phenomenon[1,8]. Most calcifications within GIST are circumscribed and patchy type. Previous episodes of bleeding or tumor necrosis with cystic degeneration may cause calcification[9-11]. In our case, the CT scan 2 years ago showed a cystic tumor with well circumscribed calcification in the stomach, but with greater thickness over the dependent part. The pre-operative CT scan with 3D reconstruction showed gastric mass with curvilinear calcification, which was identical to the operative finding. Less aggressive tumor behavior (very low risk by pathological classification) and long history of tumor presenting in this patient (more than 10 years) contributed to the development the thick calcification. The cause of the penetration was thought to be the sharp edge of the ruptured calcified cystic wall penetrating into the gastric lumen and pericolic soft tissue.

Other gastrointestinal tumors may also contain calcification. At least three types of calcification have been reported in gastric cancer: mucin pool calcifications, psammomatous calcifications, and heterotopic ossification[9,12]. In addition, four mechanisms of calcification within tumor have been suggested: (a) calcified scar tissue or granulomatous disease is engulfed by the tumor; (b) dystrophic calcification occurs within the areas of tumor necrosis; (c) calcium is deposited within the tumor as a result of a secretory function of the carcinoma; (d) metastatic calcification occurs as a result of hypercalcemia[13,14]. Mucin-forming tissues are prone to develop calcification due to the presence of mucinous material and a relatively alkaline environment, which is helpful for precipitation of calcium ions. The presence of diffuse, punctate calcifications in gastric mass is thought to be diagnostic for mucinous adenocarcinoma. Dystrophic calcification occurs in ischemic and necrotic tissue. Denatured proteins bind specifically to phosphate ions and thereafter react with calcium ions to form calcium phosphate precipitates. The relatively alkaline environment readily facilitates precipitation.

An ingested foreign body in the stomach may mimic gastric calcification. Ingested animal bone was considered initially in our case, but was excluded later due to the huge size of the calcification and because a comparison of the CT scan with an image obtained two years earlier showed that this was unlikely.

In conclusion, herein we report the first case of thick calcification from a gastric GIST with cystic degeneration penetrating into pericolic soft tissue which was successfully treated by partial gastrectomy and colectomy. For gastrointestinal tumors with thick calcification, even with benign behavior, surgical intervention should be considered for both oncological considerations and prevention of catastrophes like perforation or penetration into surrounding soft tissue.

## Consent

Written informed consent was obtained from the patient for publication of this case report and any accompanying images. A copy of the written consent is available for review by the Editor-in-Chief of this journal.

## List of abbreviations

GIST: gastrointestinal stromal tumor

## Competing interests

The authors declare that they have no competing interests.

## Authors' contributions

All authors conceived of the study, and participated in its design and coordination and helped to draft the manuscript. All authors read and approved the final manuscript.
